# P-249. Diverse populations of viable pathogenic moulds and *Candida*, including *C. auris*, are commonly recovered from air samples at two tertiary care hospitals

**DOI:** 10.1093/ofid/ofae631.453

**Published:** 2025-01-29

**Authors:** Cornelius J Clancy, Eileen Driscoll, Seong-Yoon Cho, Zachary Wilkins, Binghua Hao, Shaoji Cheng, Alexander Sundermann, Graham M Snyder, Ashley Ayres, Minh-Hong Nguyen

**Affiliations:** University of Pittsburgh, Pittsburgh, Pennsylvania; University of Pittsburgh, Pittsburgh, Pennsylvania; University of Pittsburgh, Pittsburgh, Pennsylvania; University of Pittsburgh, Pittsburgh, Pennsylvania; University of Pittsburgh Medical Center, Pittsburgh, Pennsylvania; University of Pittsburgh, Pittsburgh, Pennsylvania; University of Pittsburgh, Pittsburgh, Pennsylvania; University of Pittsburgh, Pittsburgh, Pennsylvania; UPMC, Pittsburgh, Pennsylvania; University of Pittsburgh, Pittsburgh, Pennsylvania

## Abstract

**Background:**

Airborne fungi are important nosocomial pathogens. However, there are scant data on populations of viable fungi in air from hospital environments.

**Table 1.**
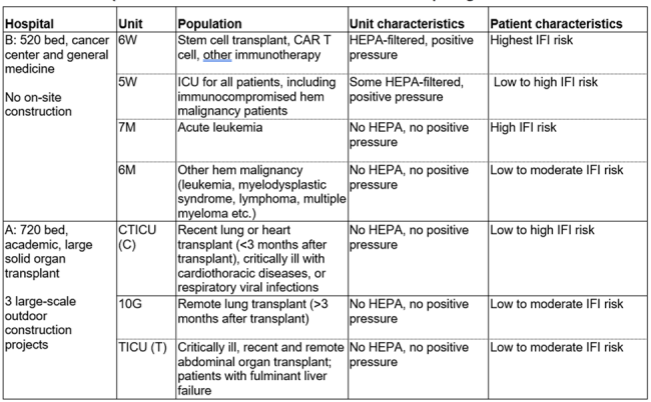
Hospitals and units included in air sampling

**Methods:**

We cultured air samples at least once/month beginning in May 2023 at 2 hospitals separated by 1.5 miles, 1 of which had new building construction [Table 1]. Air was collected with SASS 3100 (36,000 L/2 hrs onto dry filters) and SAS Super 100 (1,000 L directly onto MYE plates) samplers, at the following sites: Outside, lobby, nurse stations (NS) and rooms on units housing immunosuppressed hosts (protected (HEPA filter, positive pressure) and unprotected rooms). Processed filters (sonicated, un-sonicated) and plates were incubated at 30°C, and fungi identified by morphology and ITS sequencing.

**Table 2.**
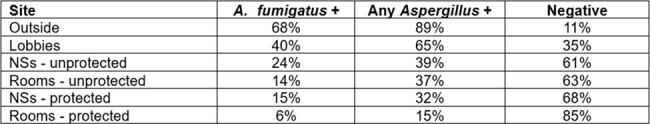
Aspergillus positive air cultures at two hospitals Data from 2 hospitals are combined and presented as % of air samples with a given result.

**Results:**

There was a hierarchy in air culture positivity for pathogenic moulds, which was similar at the 2 hospitals: outdoor cultures > lobbies > NS > unprotected rooms > protected rooms. The predominant fungus was *Aspergillus fumigatus* (68% and 6% of outdoor and protected room samples positive, respectively), followed by other *Aspergillus* spp [Table 2]. Other common moulds were *Alternaria* (48% outside +), *Cladosporium* (35% outside +)*, Fusarium* (24% outside +) and Mucorales (17% outside +, including *Rhizopus*, *Syncephalastrum racemosum* and Mucor) [Table 3]. *Candida* was cultured at similar frequencies outside and inside hospitals ((overall + (range): 7% (3%-13%)); in rank order, spp. were *C. glabrata* (n=8), *C. krusei* (6), *C. albicans* (5), *C. tropicalis* (4), *C. parapsilosis* (3), *C. auris* (1). *C. auris* was recovered from a unit that was not known to house an infected pt.

**Table 3.**
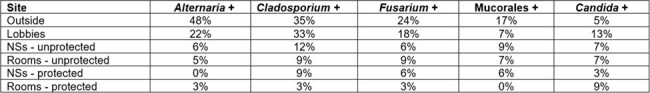
Non-Aspergillus positive air cultures at two hospitals Data from 2 hospitals are combined and presented as % of air samples with a given result.

**Conclusion:**

Potentially pathogenic fungi were commonly cultured from air samples inside and outside 2 hospitals in Pittsburgh, including within rooms housing immunosuppressed pts. Burdens were lower within hospitals than immediately outside. Fungi included moulds like *Aspergillus* spp., which are well-recognized airborne pathogens, and, surprisingly, *Candida* spp., which are not typically considered as such. Among the latter was *C. auris*, which was temporo-spatially distant from pts known to be infected at the hospitals. We are currently studying potential links between fungi recovered during surveillance and invasive fungal infections, including by whole genome sequencing.

**Disclosures:**

**Cornelius J. Clancy, MD**, Cidara: Grant/Research Support|Gilead: Honoraria|Merck: Grant/Research Support|Scynexis: Advisor/Consultant|Shionogi: Advisor/Consultant|Venatorx: Advisor/Consultant **Alexander Sundermann, DrPH, CIC, FAPIC**, OpGen: Honoraria **Graham M. Snyder, MD, SM**, Infectious Diseases Connect: Advisor/Consultant

